# Hearing Loss Diagnosis and Management in Adults with Intellectual and Developmental Disabilities

**DOI:** 10.1155/2023/6825476

**Published:** 2023-05-21

**Authors:** Alexandria Danyluk, Rafik Jacob

**Affiliations:** ^1^University of Florida, Gainesville, USA; ^2^University of Florida-Jacksonville, Department of Internal Medicine, Jacksonville, USA

## Abstract

Hearing loss is a common concern in patients with intellectual and developmental disabilities (IDD), and early detection and intervention are crucial to prevent negative impacts on communication, cognition, socialization, safety, and mental health. Despite a lack of the literature specifically focused on hearing loss in adults with IDD, there is a substantial body of research demonstrating the prevalence of hearing loss in this population. This literature review examines the diagnosis and management of hearing loss in adult patients with IDD, with a focus on primary care considerations. Primary care providers must be aware of the unique needs and presentations of patients with IDD to ensure appropriate screening and treatment. This review highlights the importance of early detection and intervention, as well as the need for further research to guide clinical practice in this patient population.

## 1. Introduction and Background

Intellectual and developmental disabilities are defined as limitations originating before the age of 22 in the domains of intellectual function, which includes learning, reasoning, and problem solving, as well as in adaptive behavior, which includes conceptual, social, and practical skills [1]. These disabilities typically originate before birth and last for the duration of a person's lifetime. Etiologies are often multifactorial, but common factors include genetics, complications during birth, infections during pregnancy or in early life, prematurity or low birth weight, and exposure to environmental toxins during pregnancy [2]. In the primary care setting, many providers report feeling uncomfortable or inadequately trained to care for individuals with IDD which raises a concern for the availability and accessibility of high quality care for patients with IDD [3].

## 2. Prevalence of Hearing Loss in Patients with IDD

A substantial body of the literature highlights the prevalence of hearing loss in patients with IDD, ranging from 24% [4] to 93% [5], depending on age and whether or not patients with down syndrome (DS) were included. One literature review showed that patients over 50 years of age with DS alone exhibited higher rates of hearing loss compared to patients over 50 with IDD, excluding DS [6]. The same applies to patients under 30, with a 7.5% [7] prevalence of hearing loss in patients with IDD versus a 42.8% prevalence in patients with DS alone [8]. In addition to the high prevalence level of hearing loss in patients with IDD, studies have also shown a positive correlation between the severity of IDD and hearing loss [9]. Finally, many studies highlight that hearing loss often goes unidentified and undertreated in patients with IDD, with previously undiagnosed hearing loss ranging from 19% to 58.7% [5, 10–13]. While hearing loss in patients without IDD can negatively impact multiple spheres of life such as cognition, communication, social contacts, safety, and occurrence of depression, patients with IDD are even more vulnerable to these consequences, making early screening, diagnosis, and treatment vital [10].

## 3. Types of Hearing Loss

### 3.1. Sensorineural Hearing Loss

Hearing loss can be divided into three primary categories, namely, conductive hearing loss (CHL), sensorineural hearing loss (SNHL), and mixed hearing loss. SNHL is the most common type of hearing loss in the general population and is due to pathologies of the cochlea, auditory, nerve, or the central nervous system, including presbycusis, congenital syndromes, infections, ototoxicity, and trauma. In a study of 106,369 athletes aged 8–70 in the International Special Olympics between 2007 and 2017, 26.9% failed hearing screenings via distortion product otoacoustic emissions (DPOAE), tympanometry, and PTA, 67.8% of which were found to have undiagnosed SNHL [14]. Various syndromes are associated with SNHL and IDD, most commonly, DS, but also CHARGE, Hurler, Hunter, and Maroteaux-Lamy. In patients with DS, SNHL is relatively low in the pediatric population, with a rate of 4.5% [15] according to one study, but increases to 65% with age [14], likely due to premature aging of the hearing system.

### 3.2. Conductive Hearing Loss

In contrast, CHL is due to any defect from the external ear through the ossicles impeding sound transmission to the cochlea, most commonly including cerumen impaction but also tympanic membrane perforation, otosclerosis, cholesteatomas, and chronic otitis media with effusion [16]. In the Special Olympics study described above, 32.2% of the athletes who failed screening via DPOAE were found to have conductive hearing loss [14]. In patients with DS, CHL is the most common cause of hearing loss with rates ranging from 53 to 88% [17] in children vs. 5–10% [18, 19] in adults, which can negatively impact speech development. Otitis media with effusion and cerumen impaction are the most prevalent etiologies of CHL patients with DS. This is likely due to T and B lymphocyte dysfunction, midfacial hypoplasia, crowed nasopharynx, enlarged adenoids, hypotonia of the tensor veli palatini muscles, and a smaller more collapsible Eustachian tube in patients with DS [20].

## 4. Identification of Hearing Loss

There is a general lack of information and evidence concerning hearing screening guidelines for patients with IDD [21]. Early and yearly objective screenings are recommended for patients with IDD or suspected cognitive delay throughout the patient's lifetime [22, 23]. Overall, the threshold for referral to audiology, otolaryngology, speech-language pathology, and genetics for these patients should be low for primary care providers so that patients can undergo developmentally appropriate multimodal hearing screens by specialists. For adults with DS, hearing loss must be evaluated as a potential contributing factor to the development of dementia or depression [18]. During yearly health evaluations in patients with IDD, providers should complete thorough head, ear, nose, throat, and neck exams [24]. This should include examining for any malformations of the auricle or ear canal, skin tags around the auricle, cleft lip/palate, hypoplastic facial structures, microcephaly, cerumen impaction, and signs of otitis media or externa. Primary care providers should examine the patient's ears with pneumatic otoscopy and may utilize the Weber/Rhinne test if feasible; however, referral is still necessary, as these methods do not fully assess hearing.

In addition to a thorough physical exam, there are various screening tests that can be performed in the primary care setting with the appropriate technology. Subjective screening methods include pure tone audiometry (PTA) and speech audiometry, whereas objective tests include tympanometry, the otoacoustic emission test (OAE), and auditory brainstem response (ABR) testing. Pure tone audiometry is a standard tool for diagnosis and characterization of hearing loss performed by primary care providers and audiologists. Typically, various frequencies are delivered to the patient via headphones (air conduction) and via a bone oscillator (bone conduction). The patient is instructed to give a signal, such as pressing a button, when the sound is heard [25]. Similarly, speech audiometry is a type of subjective hearing test that measures a person's ability to understand speech. During speech audiometry, the patient wears headphones and is presented with a series of recorded words or phrases at varying volumes, typically in a sound booth. The patient is then asked to repeat the words or phrases back to the tester. The words or phrases may be presented at different volumes to determine the softest level at which the patient can accurately repeat them, known as the speech reception threshold (SRT).

Depending on the level of cognitive delay, these subjective diagnostic tools can be challenging and necessitate ABR testing as an alternative or supplemental objective test [26]. During an ABR test, electrodes are placed on the patients' head and record the brain wave activity in response to sounds that are emitted from earphones [27]. In addition to ABR, tympanometry, an objective test that measures the movement of the eardrum in response to changes in air pressure, should be utilized in the primary care setting. The test is used to evaluate the function of the middle ear and can help diagnose conditions such as otitis media, perforated eardrum, or Eustachian tube dysfunction [28]. During tympanometry, a small probe is inserted into the ear canal. The probe contains a low-pitched sound emitting speaker and a microphone that measures the sound that is reflected back from the eardrum. The probe also contains a device that varies the air pressure in the ear canal. Finally, OAE testing, which is most often used in newborn screens, can be used in conjunction to assess hearing function or to monitor hearing loss over time. OAE objectively measures the sounds that are produced by the inner ear in response to a sound stimulus via a small probe that is placed in the ear canal [29]. The test is noninvasive, quick, and does not require any active participation from the patient.

These subjective and objective methods for hearing loss screening present various challenges for patients with IDD. Patients may have difficulty communicating their experiences or understanding instructions related to the hearing screen, thus necessitating more objective measures [30]. Patients may have behavioral issues that can make it challenging to complete a hearing screen. This may include difficulty sitting still, becoming agitated or upset, or refusing to participate in the test. Additionally, patients may have other medical conditions or sensory impairments that can complicate the hearing screen. For example, a patient with autism spectrum disorder may have difficulty tolerating the sensory input from the headphones and may need additional support to complete the test.

To overcome these challenges, it is important to first introduce the test in a way that will reduce anxiety and ensure trust between the patient and the health care provider [31]. It may be necessary to adapt the hearing screen to the patient's individual needs and provide additional support or accommodations. This may involve using alternative testing methods, providing visual aids or social stories to help the patient understand the test, or involving a caregiver or a support person in the testing process.

## 5. Treatment and Management

### 5.1. Removal of Impacted Cerumen and Regular Ear Exams

Compared to the general population, there is an increased prevalence of cerumen impaction in patients with IDD [32]. Practice guidelines recommend removing impacted cerumen when it causes symptoms or when it limits a complete clinical ear examination in the general population. A thorough history and physical should be completed to evaluate for conditions that might change the management of cerumen impaction such as nonintact tympanic membrane, ear canal stenosis, exostoses, diabetes mellitus, immunocompromised, or anticoagulant therapy, all of which may be present in patients with IDD [33]. Regular examinations, ideally every six months and no more frequent than every three months, are recommended, and treatment of impacted cerumen includes cerumenolytic agents, irrigation, and careful manual removal.

### 5.2. Tympanostomy Tubes

A recent systematic review examining appropriate management of OME in children with DS reported persisting clinical equipoise on the subject. Some studies that were included recommended conservative management in place of pressure equalizing tube placement (PET) in refractory or complicated cases, while other studies reported significant benefits including reduced complication rates with earlier PET placement in patients with down syndrome and OME. In the cases of PET failure, hearing aids may be a good alternative option [34]. As OME is more prevalent in children compared to adults with DS, limited evidence comments on the utility of tympanostomy tubes as a treatment of OME in adult patients with IDD; however, PET may be recommended in adults with refractory chronic OME.

### 5.3. Hearing Aids

Mixed success has been reported for hearing aids in patients with IDD primarily due to differing levels of disabilities. A pilot study conducted interviews with 16 adults with IDD before and 6 months after hearing aid fitting and reported improvements in distinction of sounds, auditory localization, and detection of sounds [35]. Close cooperation with the patient's caregiver as well as accessible professional support is vital for optimal fitting and success of hearing aids in patients with IDD [36].

Bone-anchored hearing aid (BAHA) placement can serve as an alternative to conventional air-conduction hearing aids in the general population as well as in patients with IDD, especially with conductive hearing loss. A case control study that observed 22 adult patients with IDD and conductive or mixed hearing loss found that after bone-anchored hearing aid placement, patients reported benefits not only in listening and learning capacities but also especially in user comfort and reduction of ear infections, as compared to conventional air-conduction hearing aids [37].

### 5.4. Cochlear Implants

Overall, variations exist in the outcomes of cochlear implants, depending on the type and level of IDD and assessment is often difficult due to poor speech perception and language skills. However, a substantial body of evidence supports the use of cochlear implants for SNHL in patients with IDD, often after failed treatment attempts with hearing aids for adults, particularly those with progressive hearing loss. Benefits in speech perception, speech intelligibility, and language development exist after cochlear implantation in patients across the age spectrum with IDD [38, 39], often tempered by degree of disability [40]. While fewer studies examined cochlear implant use in adult patients as compared to children with IDD, one study examining 13 adults with profound deafness and IDD found that patients had increased listening skills, communication skills, and self-sufficiency postimplant [41]. Reported challenges associated with cochlear implants in this patient population included managing the devices, compliance, financial barriers, and adherence to multiple follow-up appointments [42]. Another important factor that may play a key role in the outcomes of cochlear implants in adults is the structural volume loss that has been found to occur in the brain's language comprehension centers with prolonged untreated hearing loss [43].

### 5.5. Supportive Services

One 2018 study explored the benefits of speech therapy in 36 adult patients with IDD via two 3-month periods of weekly 30-minute sessions dedicated to articulation training and listening skills, with a 3-month interval in between [44]. The study reported significant improvements in speech intelligibility, receptive vocabulary, communicative initiative, and reported self-confidence, with no differences based on the level of IDD or the presence of hearing loss, implying that speech and language therapy should not be withheld from people with hearing loss [44]. The sign language often plays an important role in communication for patients with hearing loss; however, important considerations and adaptations are necessary for patients with IDD, depending on severity of disability, memory skills, and ability to physically make signs [45]. While communication adaptions must be individualized, the use of clear face to face body language within a reasonable physical distance, slow but rhythmic language, and use of pictures are important considerations.

## 6. Conclusions

The diagnosis, treatment, and management of hearing loss in patients with IDD present unique challenges due to impairments in multiple domains. Primary care providers must make special considerations with this patient population, as their presentations and needs may vary in comparison to the general population. However, there are many diagnostic tools and management options available, many of which overlap with conventional care methods that allow providers to deliver optimal care to patients with IDD and hearing loss. Many of the studies presented include pediatric data, largely due to the limited number of studies; however, early identification and treatment of hearing loss in children in the primary care setting are uniquely vital for positive longitudinal patient outcomes. Furthermore, much of the data presented from pediatric studies may be extrapolated to adult populations with IDD.

## Data Availability

No data were used to support this study.

## References

[1] American Association on Intellectual and Developmental Disabilities (2023). Defining criteria for intellectual disability. https://www.aaidd.org/intellectual-disability/definition.

[2] Centers for Disease Control and Prevention (2022). Developmental disabilities. https://www.cdc.gov/ncbddd/developmentaldisabilities/causes-and-risk-factors.html.

[3] Selick A., Durbin J., Casson I. (2022). Improving capacity to care for patients with intellectual and developmental disabilities: the value of an experiential learning model for family medicine residents. *Disability and Health Journal*.

[4] Herer G. R. (2012). Intellectual disabilities and hearing loss. *Communication Disorders Quarterly*.

[5] Evenhuis H. M., Theunissen M., Denkers I., Verschuure H., Kemme H. (2001). Prevalence of visual and hearing impairment in a Dutch institutionalized population with intellectual disability. *Journal of Intellectual Disability Research*.

[6] Bent S., McShea L., Brennan S. (2015). The importance of hearing: a review of the literature on hearing loss for older people with learning disabilities. *British Journal of Learning Disabilites*.

[7] Meuwese-Jongejeugd A., Vink M., van Zanten B. (Jan 1 2006). Prevalence of hearing loss in 1598 adults with an intellectual disability: cross-sectional population based study. *International Journal of Audiology*.

[8] Picciotti P. M., Carfì A., Anzivino R. (2017). Audiologic assessment in adults with down syndrome. *American Journal on Intellectual and Developmental Disabilities*.

[9] Määttä T., Kaski M., Taanila A., Keinänen-Kiukaanniemi S., Iivanainen M. (2023). Sensory impairments and health concerns related to the degree of intellectual disability in people with Down syndrome. *Down Syndrome Education International*.

[10] Willems M., Berlaer G., Maes L., Leyssens L., Koehler B., Marks L. (2022). Outcome of 10 years of ear and hearing screening in people with intellectual disability in Europe: a multicentre study. *Journal of Applied Research in Intellectual Disabilities*.

[11] Hild U., Hey C., Baumann U., Montgomery J., Euler H. A., Neumann K. (2008). High prevalence of hearing disorders at the Special Olympics indicate need to screen persons with intellectual disability. *Journal of Intellectual Disability Research*.

[12] McCracken W., Lumm J., Laoide-Kemp S. (2011). Hearing in athletes with intellectual disabilities: the need for improved ear care. *Journal of Applied Research in Intellectual Disabilities*.

[13] Quick N., Roush J., Erickson K., Mundy M. (2020). A hearing screening pilot study with students with significant cognitive disabilities. *Language, Speech, and Hearing Services in Schools*.

[14] Willems M., Acke F., Lannon B., Leyssens L., Maes L., Marks L. (2022). Global data on ear and hearing screening in an intellectual disability population. *American Journal on Intellectual and Developmental Disabilities*.

[15] De Schrijver L., Topsakal V., Wojciechowski M., Van de Heyning P., Boudewyns A. (2019). Prevalence and etiology of sensorineural hearing loss in children with down syndrome: a cross-sectional study. *International Journal of Pediatric Otorhinolaryngology*.

[16] Sooriyamoorthy T., De Jesus O. (2022). *Conductive Hearing Loss*.

[17] Park A. H., Wilson M. A., Stevens P. T., Harward R., Hohler N. (2012). Identification of hearing loss in pediatric patients with down syndrome. *Otolaryngology-Head and Neck Surgery*.

[18] Evenhuis H., van Zanten G., Brocaar M., Roerdinkholder W. (1992). Hearing loss in middle-age persons with Down syndrome. *American Journal on Mental Retardation*.

[19] Van Buggenhout G. J. C. M., Trommelen J. C. M., Schoenmaker A. (1999). Down syndrome in a population of elderly mentally retarded patients: genetic-diagnostic survey and implications for medical care. *American Journal of Medical Genetics*.

[20] Maris M., Wojciechowski M., Van de Heyning P., Boudewyns A. (2014). A cross-sectional analysis of otitis media with effusion in children with Down syndrome. *European Journal of Pediatrics*.

[21] Chen H. C., Wang N. M., Chiu W. C. (2014). A test protocol for assessing the hearing status of students with special needs. *International Journal of Pediatric Otorhinolaryngology*.

[22] Harlor A. D. B., Bower C. (2009). Hearing assessment in infants and children: recommendations beyond neonatal screening. *Pediatrics*.

[23] Shott S. R. (2006). Down syndrome: common otolaryngologic manifestations. *American Journal of Medical Genetics Part C: Seminars in Medical Genetics*.

[24] Oron Y., Shushan S., Ben-David N. (2015). Guidelines for ear, nose, and throat examination of adults with intellectual disabilities: report of a clinical practice application. *Journal of Policy and Practice in Intellectual Disabilities*.

[25] Us Preventive Services Task Force, Krist A. H., Davidson K. W. (2021). Screening for hearing loss in older adults: US preventive services task force recommendation statement. *JAMA*.

[26] Trudeau S., Anne S., Otteson T., Hopkins B., Georgopoulos R., Wentland C. (2021). Diagnosis and patterns of hearing loss in children with severe developmental delay. *American Journal of Otolaryngology*.

[27] Chien C. H., Tu T. Y., Shiao A. S. (2008). Prediction of the pure-tone average from the speech reception and auditory brainstem response thresholds in a geriatric population. *ORL*.

[28] Baldwin R. L., Carmichael L. (1983). Practical tympanometry and stapedial reflex testing in office practice. *Alabama Medicine*.

[29] Kemp D. T. (1978). Stimulated acoustic emissions from within the human auditory system. *Journal of the Acoustical Society of America*.

[30] Nightengale E. E., Wolter-Warmerdam K., Yoon P. J., Daniels D., Hickey F. (2020). Behavioral audiology procedures in children with down syndrome. *American Journal of Audiology*.

[31] Sharby N., Martire K., Iversen M. D. (2015). Decreasing health disparities for people with disabilities through improved communication strategies and awareness. *International Journal of Environmental Research and Public Health*.

[32] Crandell C. C., Roeser R. J. (1993). Incidence of excessive/impacted cerumen in individuals with mental retardation: a longitudinal investigation. *American Journal of Mental Retardation: American Journal of Mental Retardation*.

[33] Roland P. S., Smith T. L., Schwartz S. R. (2008). Clinical practice guideline: cerumen impaction. *Otolaryngology-head and neck surgery: Official Journal of American Academy of Otolaryngology-Head and Neck Surgery*.

[34] Sait S., Alamoudi S., Zawawi F. (2022). Management outcomes of otitis media with effusion in children with down syndrome: a systematic review. *International Journal of Pediatric Otorhinolaryngology*.

[35] Meuwese-Jongejeugd A., Verschuure H., Evenhuis H. M. (2007). Hearing aids: expectations and satisfaction of people with an intellectual disability, a descriptive pilot study. *Journal of Intellectual Disability Research*.

[36] Blazer D. G., Domnitz S., Liverman C. T. (2016). *Hearing Health Care for Adults: Priorities for Improving Access and Affordability*.

[37] Kunst S. J. W., Hol M. K., Cremers C. W., Mylanus E. A. (2007). Bone-anchored hearing aid in patients with moderate mental retardation: impact and benefit assessment. *Otology & Neurotology*.

[38] Casazza G., Meier J. D. (2017). Evaluation and management of syndromic congenital hearing loss. *Current Opinion in Otolaryngology & Head and Neck Surgery*.

[39] van Nierop J. W. I., Huinck W. J., Pennings R. J. E., Admiraal R. J. C., Mylanus E. A. M., Kunst H. P. M. (2016). Patients with Pendred syndrome: is cochlear implantation beneficial?. *Clinical Otolaryngology*.

[40] Lee Y. M., Kim L. S., Jeong S. W., Kim J. S., Chung S. H. (2010). Performance of children with mental retardation after cochlear implantation: speech perception, speech intelligibility, and language development. *Acta Oto-Laryngologica*.

[41] Filipo R., Bosco E., Mancini P., Ballantyne D. (2004). Cochlear implants in special cases: deafness in the presence of disabilities and/or associated problems. *Acta Oto-Laryngologica*.

[42] Zaidman-Zait A., Curle D., Jamieson J. R., Chia R., Kozak F. K. (2015). Cochlear implantation among deaf children with additional disabilities: parental perceptions of benefits, challenges, and service provision. *Journal of Deaf Studies and Deaf Education*.

[43] Sun Z., Seo J. W., Park H. J. (2021). Cortical reorganization following auditory deprivation predicts cochlear implant performance in postlingually deaf adults. *Human Brain Mapping*.

[44] Terband H., Coppens-Hofman M. C., Reffeltrath M., Maassen B. A. M. (2018). Effectiveness of speech therapy in adults with intellectual disabilities. *Journal of Applied Research in Intellectual Disabilities*.

[45] van Dijk R., Nelson C., Postma A., van Dijk J. (2010). Deaf children with severe multiple disabilities: etiologies, intervention, and assessment. *The Oxford handbook of deaf studies, language, and education*.

